# Lack of Vesicular Zinc Does Not Affect the Behavioral Phenotype of Polyinosinic:Polycytidylic Acid-Induced Maternal Immune Activation Mice

**DOI:** 10.3389/fnbeh.2022.769322

**Published:** 2022-02-22

**Authors:** Katy Celina Sandoval, Sarah E. Thackray, Alison Wong, Nicole Niewinski, Colten Chipak, Suhkjinder Rehal, Richard H. Dyck

**Affiliations:** ^1^Department of Psychology, University of Calgary, Calgary, AB, Canada; ^2^Hotchkiss Brain Institute (HBI), University of Calgary, Calgary, AB, Canada; ^3^Alberta Children’s Hospital Research Institute (ACHRI), University of Calgary, Calgary, AB, Canada; ^4^Department of Cell Biology and Anatomy, University of Calgary, Calgary, AB, Canada

**Keywords:** vesicular zinc, maternal immune activation, autism spectrum disorders, schizophrenia, polyI:C, behavior, ZnT3, *SLC30A3*

## Abstract

Zinc is important in neural and synaptic development and neuronal transmission. Within the brain, zinc transporter 3 (ZnT3) is essential for zinc uptake into vesicles. Loss of vesicular zinc has been shown to produce neurodevelopmental disorder (NDD)-like behavior, such as decreased social interaction and increased anxiety- and repetitive-like behavior. Maternal immune activation (MIA) has been identified as an environmental factor for NDDs, such as autism spectrum disorders (ASDs) and schizophrenia (SZ), in offspring, which occurs during pregnancy when the mother’s immune system reacts to the exposure to viruses or infectious diseases. In this study, we investigated the interaction effect of a genetic factor [ZnT3 knockout (KO) mice] and an environmental factor (MIA). We induced MIA in pregnant female (dams) mice during mid-gestation, using polyinosinic:polycytidylic acid (polyI:C), which mimics a viral infection. Male and female ZnT3 KO and wild-type (WT) offspring were tested in five behavioral paradigms: Ultrasonic Vocalizations (USVs) at postnatal day 9 (P9), Open Field Test, Marble Burying Test, three-Chamber Social Test, and Pre-pulse Inhibition (PPI) in adulthood (P60–75). Our results indicate that loss of vesicular zinc does not result in enhanced ASD- and SZ-like phenotype compared to WT, nor does it show a more pronounced phenotype in male ZnT3 KO compared to female ZnT3 KO. Finally, MIA offspring demonstrated an ASD- and SZ-like phenotype only in specific behavioral tests: increased calls emitted in USVs and fewer marbles buried. Our results suggest that there is no interaction between the loss of vesicular zinc and MIA induction in the susceptibility to developing an ASD- and SZ-like phenotype.

## Introduction

Brain development is a complex process that is influenced by genetic and environmental factors. The development of the central nervous system, which begins in the early embryonic stages, includes various critical periods of vulnerability, at which point alterations to the fetal environment can result in structural and functional abnormalities in offspring organs, including the brain (Rice and Barone, 2000; Schlotz and Phillips, 2009). This could lead to increased susceptibility to diseases and psychiatric disorders later in life as physiological changes may occur in the process (Rice and Barone, 2000; Schlotz and Phillips, 2009). For instance, during pregnancy, maternal immune activation (MIA) can lead to changes in the fetal environment, contributing to the disruption of brain development in exposed offspring (Meyer, 2014). Zinc deficiency is another risk factor that has been identified as a contributor to neurodevelopmental disorders (NDDs), such as autism spectrum disorders (ASDs) and schizophrenia (SZ) (Yasuda et al., 2011; Grabrucker, 2013; Nuttall, 2017; Ha et al., 2018; Joe et al., 2018). Zinc is an essential component of the structure and functioning of the brain. It plays a role in the development of neurons and synaptic connections, as well as in neural transmission (Sandstead et al., 2000). Within the brain, zinc transporter 3 (ZnT3) is important for the uptake of zinc into vesicles and it is expressed in zinc-enriched areas such as the cerebral cortex, amygdala, and hippocampus [as depicted by Cole et al. (1999) using modifications of Timm’s silver-sulfide stain].

Zinc concentration in the brain increases with age and remains constant in adulthood (Takeda, 2001). Dysregulation of zinc homeostasis has the potential to modify the functioning of neurotransmitter receptors and second-messenger systems, potentially causing brain dysfunctions and neurological diseases (Takeda, 2000; Nakashima and Dyck, 2008). Loss of vesicular zinc has been shown to produce NDD-like behavior, including decreased social interaction and preference for social novelty; decreased time spent in the center of the open field test; increased repetitive behavior in the marble burying test; and age-related cognitive decline, as shown by deficits in the novel object preference test, the Morris water task, and T-maze test (Cole et al., 1999; Yoo et al., 2016; McAllister and Dyck, 2017). Dietary zinc deficiency has also been associated with different conditions including neural development disorders, impaired immunity, and degenerative diseases (Yasuda et al., 2011; McAllister and Dyck, 2017).

Autism spectrum disorders and SZ are associated to prenatal risk factors, such as MIA (Canetta and Brown, 2012; Canetta et al., 2016; Scola and Duong, 2017). ASDs are defined as NDDs characterized by key behavioral features: social impairments, difficulties communicating, stereotyped behaviors, and abnormal responses to sensory stimulation (Patterson, 2006; Theoharides and Zhang, 2011; Yoo et al., 2016). According to the Public Health Agency of Canada, in 2018, 1 in 66 children and youth have been diagnosed with ASDs, and it is predominantly diagnosed in males (Public Health Agency of Canada, 2018). In humans, ASDs are usually noticed in the first or second year of life, indicating that prenatal and/or early postnatal development may be critical (Koh et al., 2014).

As previously mentioned, genetic risk is thought to be the leading cause of ASDs. More than 500 genes may be involved (Parellada et al., 2014; Ronemus et al., 2014). One of the well-known genes associated with ASDs is *SHANK3*. Mutations of this gene have been found in people with ASDs, making it a candidate gene to study for these disorders (Fourie et al., 2018). *SHANK3* is involved in synapse formation and synaptic transmission, providing support to organize other proteins at the synapse (Koh et al., 2014; Arons et al., 2016; Tao-Cheng et al., 2016). Interestingly, *SHANK3* activation and function requires zinc, and an endogenous source of free zinc for *SHANK3* modulation is the release of zinc from synaptic vesicles (Koh et al., 2014; Arons et al., 2016; Ha et al., 2018).

Schizophrenia is a psychotic disorder that affects approximately 1% of the population worldwide (Meyer and Feldon, 2012). It is defined by positive symptoms (e.g., hallucinations and/or delusions), negative symptoms (e.g., anhedonia), and cognitive deficits associated with the positive and negative dichotomy (e.g., attention and memory deficits) (Missault et al., 2014). SZ is usually noticed during adolescence or early adulthood (Meyer and Feldon, 2009). The disruptions in behavioral, mental, and emotional functions are believed to be a product of genetic and environmental factors in brain development early in life and peri-adolescence (Meyer and Feldon, 2009).

A gene that has been associated with SZ is *SLC30A3* (ZnT3) expressed in the glutamate synapse in the hippocampus and cerebral cortex; it has been shown to be downregulated in three SZ patient cohorts (Maycox et al., 2009; Perez-Becerril et al., 2014, 2016). The findings suggested that ZnT3-gene is a SZ susceptibility gene that could have sex-dependent effects, impacting females more than males. Another gene that has been associated with SZ is *SHANK3*, the same gene previously mentioned to be associated with ASDs (Arons et al., 2016; Zhou et al., 2016).

Zinc deficiency during pregnancy has been shown to increase the risk of impairments in offspring (Vela et al., 2015). There is clinical evidence that zinc deficiency may impact brain development, as low levels of serum zinc are commonly observed in patients with ASDs and SZ (Grabrucker, 2013; Joe et al., 2018).

Zinc deficiency, from diet in humans and ZnT3 KO in mice, has been shown to trigger a suppressed immune response to pathogens making animals with zinc deficiency more susceptible to infections (Fukada and Kambe, 2018). A study by Yoo et al. (2016) used ZnT3 KO mice to study the role of vesicular zinc in ASDs. Their results suggest that ZnT3 KO leads to a sex-dependent autistic-like phenotype (Yoo et al., 2016).

Maternal immune activation has been modeled in rodents by exposure to a pathogen, which triggers an immune response in the mother. Epidemiological studies looking at the effects of MIA have shown a recurrent link with NDDs in adult offspring (Fortier et al., 2007; Atladóttir et al., 2010; Bitanihirwe et al., 2010; Vuillermot et al., 2012; Zhang et al., 2012). A well-known model of viral-like immune activation is achieved by injection of polyinosinic:polycytidylic acid (polyI:C), which mimics a viral infection as a synthetic viral-like double stranded RNA (dsRNA) (Fortier et al., 2004). Injections of polyI:C in rodents have been shown to induce sickness behavior, such as reduced appetite, decreased body weight, and increased body temperature (Fortier et al., 2004; Ratnayake et al., 2014).

Polyinosinic:polycytidylic acid has been associated with Toll-like receptor-3 (TLR3), a receptor that is specific to dsRNA viral infection, as it activates cytokines (including interleukin-1β, interleukin-6, and tumor necrosis factor-α), causing an inflammatory response (Fortier et al., 2004; Cunningham et al., 2007). It is not surprising that the use of polyI:C in rodents increases the level of pro-inflammatory factors, such as cytokines, due to its interaction with TLR3 (Fortier et al., 2004; Cunningham et al., 2007; Ratnayake et al., 2014). Cytokines are small proteins that are involved in cell-to-cell communication produced by immune cells in response to inflammation and are involved in the regulation of neurodevelopment processes (Scola and Duong, 2017).

Maternal immune activation models exhibit disturbances in a variety of brain regions, including the hippocampus, prefrontal cortex, insula, cingulate cortex, mid-temporal lobe, and parietal lobe (Spann et al., 2018). These brain regions are involved in deciphering emotions, behavioral reactivity, attention, and learning and memory (Scola and Duong, 2017; Spann et al., 2018). Non-human animal studies have identified behavioral abnormalities in MIA exposed offspring using various behavioral tasks relevant to NDD-like symptoms: anxiety (open-field test, elevated plus maze), communication [ultrasonic vocalizations (USVs), olfactory sensitivity], social interaction (three-chamber social test, social recognition), repetitive behavior (marble burying test, self-grooming), and sensory stimuli sensitivity [pre-pulse inhibition (PPI), latent inhibition] (Meyer et al., 2005; Crawley, 2007; Smith et al., 2007; Malkova et al., 2012; Ratnayake et al., 2014; Kim et al., 2017; Mueller et al., 2021).

To our knowledge, it is unknown whether or not there is an interaction between an environmental factor (MIA) and the genetic factor of ZnT3 deletion. To elucidate the behavioral consequences following MIA exposure, offspring underwent a battery of behavioral assays to assess core symptoms associated with ASD- and SZ-like symptoms, as well as comorbid features, often observed in human patients: USVs, open field, marble burying, three-chamber social test and PPI. Based on previous studies, we hypothesized that offspring of a polyI:C exposed mothers would demonstrate an NDD-like phenotype compared to the control offspring. Additionally, we expected that this phenotype would be more severe in ZnT3 KO mice than in wild-type (WT) mice. More specifically, we expected to observe either an increased or decreased number and length of USVs in pups. In adult mice, it was anticipated that they would have decreased time spent in the center of the open field test—indicating increased anxiety-like behavior—as well as less distance traveled in the open field, increased stereotyped behavior in the marble burying test, decreased social interaction in the three-chamber social test, and low inhibition in the PPI test. Furthermore, we hypothesized that this phenotype would be more pronounced in male ZnT3 KO mice compared to female ZnT3 KO mice. Lastly, we hypothesized that there would be an interaction effect between genotype and treatment in which case ZnT3 KO offspring of polyI:C-injected mothers would show the more severe NDD-like phenotype.

## Materials and Methods

### Animals

All procedures were approved by the Life and Environmental Sciences Animal Care Committee at the University of Calgary and conformed to the guidelines established by the Canadian Council on Animal Care. Male and female C57BL/6 × 129Sv mice heterozygous for the ZnT3-coding gene (*slc30A3*) were paired and housed in standard cages (28 cm × 17.5 cm × 12 cm, bedding, nesting material and a house as an enrichment object). Offspring, both male and female, were housed with the mother until postnatal day 21 (P21), after which they were weaned and housed in standard cages with 2–5 littermates of the same sex. They were kept on a 12 h light/dark cycle with lights on at 7 a.m. at an ambient room temperature of 22°C, and food and water available *ad libitum*.

### Experimental Design

We generated seven cohorts of offspring for this experiment. In total, 71 heterozygous (ZnT3+/−) females were paired with 57 heterozygous males, but only 45 were impregnated and gave birth. From the 45 pregnant females, 7 dams did not deliver live pups, and 1 dam had complications during delivery, resulting in a total of 37 viable litters.

To determine the beginning of gestation, the appearance of a seminal plug was considered embryonic day 0.5 (E0.5). At E12.5, dams were injected with polyI:C (20 mg/kg; Sigma-Aldrich, St. Louis, Mo, United States), which was dissolved in 0.9% saline and administered *via* intraperitoneal (i.p.) injection. Control females were given an equivalent volume of 0.9% saline (0.001 mL/g), also *via* i.p. injection. That same day (E12.5) male breeders were removed from the cage and the dams were single housed until birth. The body weights of dams were recorded on a daily basis until E16.5 to confirm that the polyI:C induced an acute response, identified by a decrease in weight, 24-h post-injection (Fortier et al., 2004).

**FIGURE 1:**
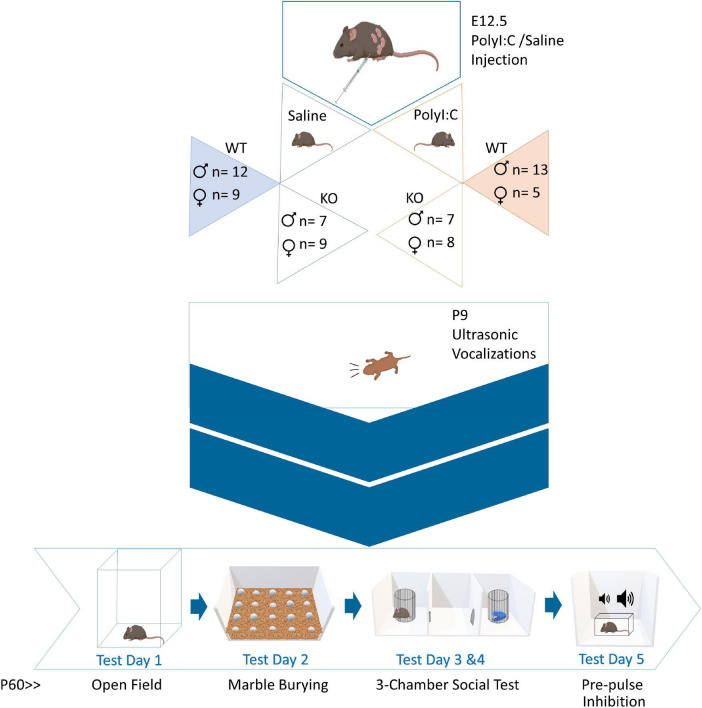
Experimental timeline. Pregnant dams were administered polyinosinic:polycytidylic acid (polyI:C) at embryonic day 12.5. Female and male, wild-type (WT) and zinc transporter 3 knockout (ZnT3 KO) offspring underwent five behavioral tests. The first behavioral test was completed on P9. The other tests were done in adulthood, between P60-P75, across five consecutive days.

Offspring were genotyped after being weaned in order to determine which mice to use for behavioral testing. In this case, only WT and ZnT3 KO male (WT-Saline: *n* = 12; KO-Saline: *n* = 7; WT-PolyI:C: *n* = 13; KO-PolyI:C: *n* = 7) and female (WT-Saline: *n* = 9; KO-Saline: *n* = 9; WT-PolyI:C: *n* = 5; KO-PolyI:C: *n* = 8) offspring were selected for testing. Ear tissue sample was taken from mice to extract DNA using proteinase K. Polymerase chain reaction (PCR) was used to amplify DNA, using primers oIMR3663 (mutant), oIMR3693 (WT), oIMR3694 (common). To determine the alleles contained in the sample, we ran gel electrophoresis accompanied by positive and negative controls. For a diagram of the experimental timeline, see Figure 1.

### Behavioral Assessments

For the following tests, experimental mice were habituated to the test room for 30-min prior to each testing day. All tests were conducted during the light phase of the light/dark cycle between 10 a.m. and 5 p.m.

#### Ultrasonic Vocalizations

At P9, the dams were removed from the nest and placed in a clean holding cage while their pups were left in their home cage. The home cage, containing the pups, was moved to the testing room. Following habituation, pups were removed from the home cage, one at a time, isolated from their siblings and placed in a Plexiglas recording chamber (28 cm × 17.5 cm × 12 cm). The test was performed in a room with the lights turned off. USVs were recorded for 4-min using an UltraSoundGate 116Hm microphone (Avisoft Bioacoustics, Berlin, Germany) and collected using Avisoft Recorder USGH (Kim et al., 2017). After the USV recording, pups were placed in a separate holding cage to avoid re-testing the same pup.

To avoid any stress in the mother and offspring due to foreign olfactory cues, gloves were changed after handling each litter. Additionally, gloved hands were rubbed in the cage bedding of the home cage for a few seconds, prior to handling the pups to ensure the transfer of the litter smell to the gloves. The recording cage was cleaned with 70% ethanol after each litter.

The recorded USV calls were scored using DeepSqueak 2.6.1 software (Coffey et al., 2019). DeepSqueak is a free software that runs through MATLAB (R2018b, Natick, MA, United States). The software was used to determine the number of calls made by the pups and the length of their calls.

#### Open Field Test

To assess anxiety-like behavior, mice were placed individually near the center of an arena (40 cm × 40 cm × 40 cm) containing approximately 1.3 cm bedding. We have found that the addition of bedding, which is not typical, reduces stress in the mice and has allowed us to probe for any stereotypies. Movement was recorded for a period of 10-min (Kim et al., 2017). We used an overhead camera (Basler acA1300-60mg GigE, Basler AG, Berlin, Germany) fixed to the ceiling above the arenas, or a Sony Handycam HDR-SR8 camera for the 2nd cohort. The total distance traveled, and the total time spent in the center of the field (20 cm × 20 cm) were measured using EthoVision XT 14 software (Noldus Information Technology Inc., Leesburg, VA, United States).

The arena was cleaned with 70% ethanol after each mouse. Mice were tested by sex group, and the bedding was changed between male and female mice.

#### Marble Burying Test

To measure repetitive behavior, mice were placed in a cage (28 cm × 17.5 cm × 12 cm) containing 4 cm of bedding. We used 20 identical black-painted glass marbles placed in an equidistant 4 × 5 array on top of the bedding. Mice were left in the cage for a duration of 10-min, after which they were placed back into their home cage (Kim et al., 2017). The number of marbles buried in 10-min was counted and converted to a three-level scale: if the marble was not buried, it was given a score of 0; if it was 50% buried or less it was given a score of 0.5; and if it was buried more than 50% buried a score of 1 was given. The sum of these scores was divided by 20 to obtain the ratio of marbles buried.

The cage with bedding and the marbles were cleaned with 70% ethanol after each mouse. Mice were tested by sex group, and separate cages were used for male and female mice.

#### Three-Chamber Social Test

Social interaction was assessed using the three-chamber test across 2-days. On the first day, mice were placed individually in the three-chamber arena (40 cm × 60 cm × 22 cm partitioned equally lengthwise) and left to freely explore all chambers for 10-min. The right and left chambers held an empty wire-mesh cylinder (8.5 cm diameter × 9.5 cm) (Kim et al., 2017). Therefore, this exploration allowed for the evaluation of a mouse’s preferred cylinder and chamber. The next day, the doors to the right and left chambers were initially blocked, and the mouse was placed in the middle chamber for 5-min (habituation). After this period, the doors were opened to allow free exploration in all chambers for 10-min. The previously determined preferred side had a novel object (a mini stapler) in the mesh cage, and the other side had a novel conspecific, a heterozygous mouse of the same sex and age as the experimental mouse, in the mesh cage. In the cases where a mouse did not have a preference, cylinders were placed randomly.

An overhead camera (Basler acA1300-60mg GigE, Basler AG, Germany) was used to record the total time spent exploring each mesh cage containing the novel conspecific/novel object. EthoVision XT 14 software (Noldus Information Technology Inc., Leesburg, VA, United States) was used to automatically assess the total time spent exploring each mesh cage. The social index was calculated using the time spent exploring mesh cages: $\frac{n\,o\,v\,e\,l\,c\,o\,n\,s\,p\,e\,c\,i\,f\,i\,c}{n\,o\,v\,e\,l\,c\,o\,n\,s\,p\,e\,c\,i\,f\,i\,c\,n\,o\,v\,e\,l\,\text{õ}\,b\,j\,e\,c\,t}$.

The arena was cleaned with 70% ethanol after each mouse. Mice were tested by sex group, and the bedding was changed between male and female mice.

#### Pre-pulse Inhibition

Sensory-motor gating was assessed using PPI of the acoustic startle response. This test involves the presentation of a lower intensity sound prior to the acoustic startle stimulus and the measurement of the startle response. The mice were individually placed in an isolation chamber (5 cm × 10 cm × 5 cm), which includes a force sensor to detect the movement of the mouse. The startle response was measured and analyzed using a SM100SP Startle Monitor system (Hamilton-Kinder LLC, San Diego, CA, United States).

The session began with a 5-min acclimatization period with background white noise (65 dB) and proceeded with four 115 dB, 40 ms sound bursts—which were not included in the analysis as they measure the baseline of the acoustic startle response. The session included six of each of the following trial types for a total of 18 trials: 20 ms pre-pulse stimuli at 70 dB (PPI 5 dB higher than background), 75 dB (PPI 10 dB higher than background), and 85 dB (PPI 20 dB higher than background) (Thackray et al., 2017). Each session lasted approximately 18-min. Percent PPI was calculated using the formula: $\frac{p\,u\,l\,s\,e-p\,r\,e\,p\,u\,l\,s\,e}{p\,u\,l\,s\,e}\,100$.

The chamber was cleaned with 70% ethanol after each mouse. Mice were tested by sex group.

### Statistical Analysis

Statistical analyses were conducted using IBM SPSS Statistics (version 25). A 2-way Analysis of Variance (ANOVA) with [Genotype (WT vs. ZnT3 KO) × Treatment (Saline vs. PolyI:C)] as factors was performed to analyze each behavioral test, unless otherwise specified. A separate ANOVA was run for each sex (male and female). A critical alpha of *p* < 0.05 was used to assess statistical significance, and significant interactions were followed up with (Bonferroni-corrected) *post-hoc* tests. GraphPad Prism 8 software was used to create all the graphs presented in the section “Results.”

## Results

### Weight of Mothers and Litter Size

**FIGURE 2:**
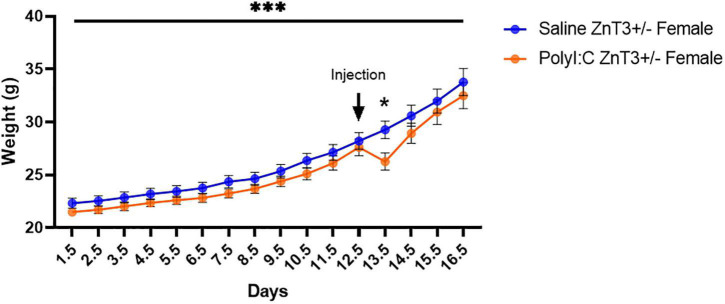
Body weight of ZnT3+/– dams, recorded from gestational day 1–16. A significant body weight decrease was observed 24-h after polyinosinic:polycytidylic acid (polyI:C) administration (Day 13). Error bars depict SEM, *p < 0.05, ***p < 0.001.

A repeated-measures ANOVA [Treatment (Saline vs. PolyI:C) × Days (day 1.5–day 16.5)] was performed to assess the weight of the dams during pregnancy. Sphericity was violated according to Mauchly’s Test of Sphericity, χ^2^(119) = 1040.171, *p* < 0.001, and therefore, a Huynh-Feldt correction was used (ε = 0.083). All pregnant females (dams) increased in body weight as pregnancy progressed [*F*_(1.228, 34.373)_ = 146.500, *p* < 0.001], with no difference in weight progression between saline- and polyI:C-injected dams [*F*_(1.228, 34.373)_ = 0.848, *p* = 0.386]. We previously hypothesized that dams injected with polyI:C would demonstrate a loss of weight 24-h post-injection (day 13.5), and saline-injected dams would continue gaining weight consistently. *A priori* comparison revealed that the weight significantly decreased in polyI:C-injected dams by an average of 2.956 g [*F*_(1,__28)_ = 5.877, *p* = 0.022] at day 13.5 (Figure 2). Furthermore, 72% of the dams from the saline group gave birth to viable litters, but only 49% of polyI:C-injected dams gave birth to viable litters. However, litter size did not differ between the saline-injected (7.40 ± 2.542) and polyI:C-injected (7.29 ± 2.544) groups [*t*(35) = 0.126, *p* = 0.900].

### Ultrasonic Vocalizations

We determined the latency to emit the first call, counted the number of USV calls emitted by pups, and measured the average length of calls. Means ± SD values can be found in Supplementary Table 1.

#### Latency

**FIGURE 3:**
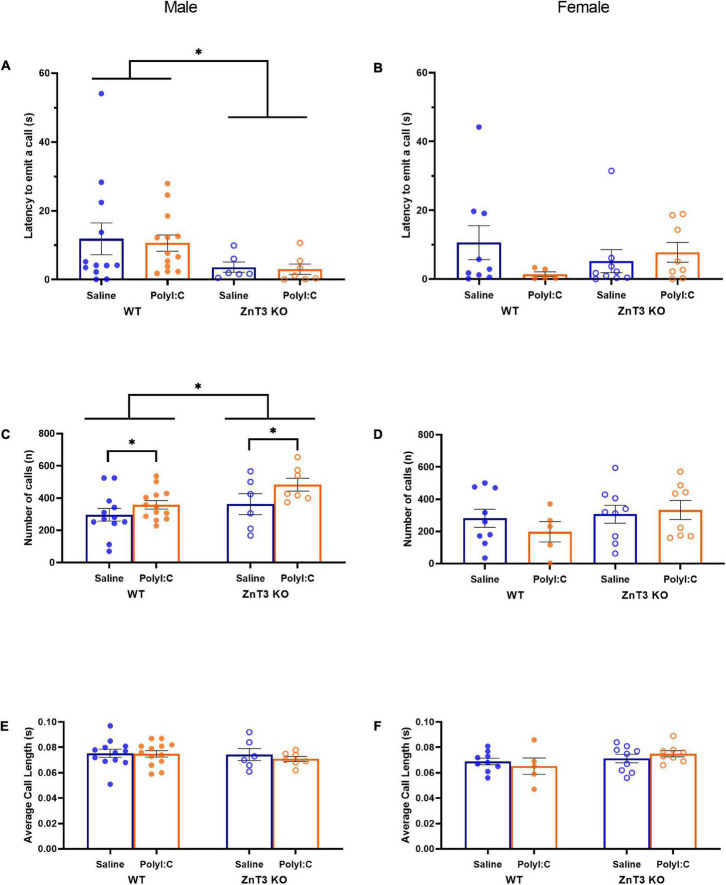
Ultrasonic vocalizations (USVs) by postnatal day 9 pups. Latency to emit a call **(A,B)**, number of calls **(C,D)** and average call length **(E,F)** were measured. **(A)** Male zinc transporter 3 knockout (ZnT3 KO) offspring took less time to emit their first call compared to wild-type (WT) offspring. No effect of polyinosinic:polycytidylic acid (polyI:C) treatment was observed. **(B)** In female offspring, no significant difference was observed. **(C)** Male maternal immune activation (MIA)-offspring emitted more calls than saline-injected offspring and male ZnT3 KO offspring emitted more calls than WT offspring. **(D)** No significant difference was observed in female offspring. **(E)** Male offspring did not demonstrate a statistically significant difference between treatments or genotypes. **(F)** Female offspring did not demonstrate statistically significant differences between treatments or genotypes. Error bars depict SEM, *p < 0.05.

In males, there was a significant difference between genotypes [main effect of genotype: *F*_(1,__34)_ = 4.695, *p* = 0.037; genotype × treatment interaction: *F*_(1,__34)_ = 0.008, *p* = 0.931] in which ZnT3 KO male offspring emitted their first call sooner than their WT counterparts. There was no significant difference between treatments [main effect of treatment: *F*_(1,__34)_ = 0.061, *p* = 0.807] (Figure 3A).

In females, there was no significant difference between genotypes or treatments on the latency to emit the first call [main effect of genotype: *F*_(1,__27)_ = 0.016, *p* = 0.899; main effect of treatment: *F*_(1,__27)_ = 0.717, *p* = 0.404; genotype × treatment interaction: *F*_(1,__27)_ = 2.253, *p* = 0.145] (Figure 3B).

#### Number of Calls

In males, there was a significant difference between the two genotypes in the number of calls emitted [main effect of genotype: *F*_(1,__34)_ = 5.165, *p* = 0.029; genotype × treatment interaction: *F*_(1,__34)_ = 0.498, *p* = 0.485], with ZnT3 KO male offspring emitting a greater number of calls than WT offspring. There was also a significant difference between the two treatments observed [*F*_(1,__34)_ = 4.734, *p* = 0.037], in which MIA-offspring emitted more calls than male offspring of saline-injected mothers (Figure 3C).

In females, there was no significant difference between genotypes or treatments for the number of calls emitted [main effect of genotype: *F*_(1,__27)_ = 0.491, *p* = 0.490; main effect of treatment: *F*_(1,__27)_ = 0.029, *p* = 0.865; genotype × treatment interaction: *F*_(1,__27)_ = 0.216, *p* = 0.646] (Figure 3D).

#### Calls Length

In males, there was no significant difference between genotypes or treatments for the number of calls emitted [main effect of genotype: *F*_(1,__34)_ = 0.569, *p* = 0.456; main effect of treatment: *F*_(1,__34)_ = 0.326, *p* = 0.572; genotype × treatment interaction: *F*_(1,__34)_ = 0.193, *p* = 0.663] (Figure 3E).

In females, there was no significant difference between genotypes or treatments for the number of calls emitted [main effect of genotype: *F*_(1,__27)_ = 1.499, *p* = 0.231; main effect of treatment: *F*_(1,__27)_ = 0.488, *p* = 0.491; genotype × treatment interaction: *F*_(1,__27)_ = 0.325, *p* = 0.573] (Figure 3F).

### Open Field

#### Distance Traveled

**FIGURE 4:**
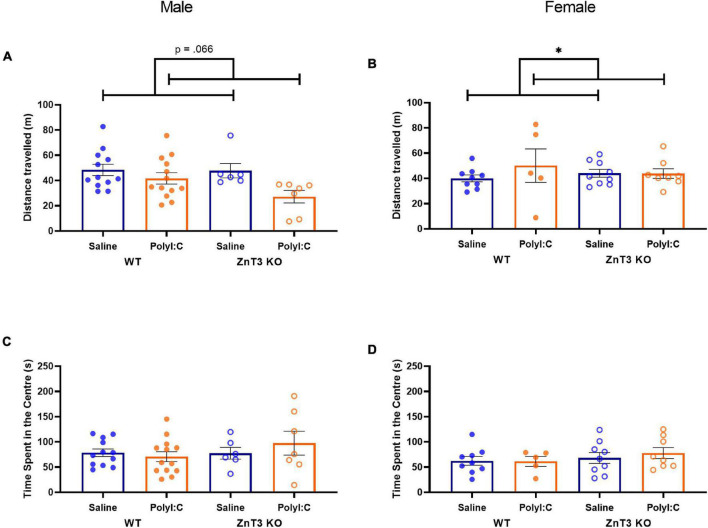
Distance traveled **(A,B)** and time spent in the center **(C,D)** in the open field test. **(A)** Male maternal immune activation (MIA)-offspring tended to travel shorter distances than male offspring of saline-injected mothers, though the difference was not significant. **(B)** Female MIA-offspring traveled greater distances than female offspring from saline-injected mothers. **(C,D)** No significant differences were observed in the time spent in the center between treatment and genotype, in either male or female offspring. Error bars depict SEM, *p < 0.05.

In males, there was no significant difference between genotypes or treatments on the distance traveled [main effect of genotype: *F*_(1,__34)_ = 0.434, *p* = 0.514; main effect of treatment: *F*_(1,__34)_ = 3.617, *p* = 0.066; genotype × treatment interaction: *F*_(1,__34)_ = 0.306, *p* = 0.584] (Figure 4A).

In females, there was a statistically significant interaction between treatments and genotypes on the distance traveled [*F*_(1,__27)_ = 4.569, *p* = 0.042]. Follow-up *t*-tests did not identify significant effects of polyI:C treatment in either genotype [WT: *t*(4.764) = −1.961, *p* = 0.110; KO: *t*(15) = 0.007, *p* = 0.994]. No significant difference was observed between genotypes [*F*_(1,__27)_ = 1.530, *p* = 0.227]. There was a significant main effect of treatment [*F*_(1,__27)_ = 4.533, *p* = 0.043] in which MIA-offspring traveled longer distances (Figure 4B). Means ± SD can be found in Supplementary Table 1.

#### Time Spent in the Center

In males, there was no significant difference between genotypes or treatments on the time spent in the center of the open field [main effect of genotype: *F*_(1,__34)_ = 0.963, *p* = 0.333; main effect of treatment: *F*_(1,__34)_ = 0.219, *p* = 0.643; genotype × treatment interaction: *F*_(1,__34)_ = 1.099, *p* = 0.302] (Figure 4C).

In females, there was no significant difference between genotypes or treatments on the time spent in the center of the open field [main effect of genotype: *F*_(1,__27)_ = 0.730, *p* = 0.400; main effect of treatment: *F*_(1,__27)_ = 0.495, *p* = 0.488; genotype × treatment interaction: *F*_(1,__27)_ = 0.341, *p* = 0.564] (Figure 4D).

### Marble Burying

**FIGURE 5:**
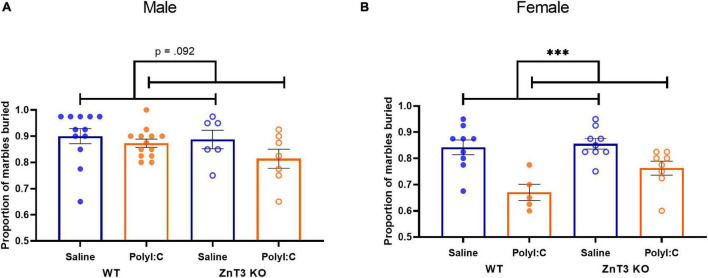
Marble burying test. **(A)** Male maternal immune activation (MIA)-offspring tended to bury fewer marbles compared to saline-offspring, though the difference was not significant. **(B)** Female MIA-offspring buried fewer marbles compared to saline-offspring. Error bars depict SEM, ^***^p < 0.001.

In males, there was no significant difference between genotypes or treatments on the proportion of marbles buried [main effect of genotype: *F*_(1,__34)_ = 1.524, *p* = 0.225; main effect of treatment: *F*_(1,__34)_ = 3.007, *p* = 0.092; genotype × treatment interaction: *F*_(1,__34)_ = 0.643, *p* = 0.428] (Figure 5A).

In females, there was no significant difference between the two genotypes on the proportion of marbles buried [main effect of genotype: *F*_(1,__27)_ = 3.148, *p* = 0.087; genotype × treatment interaction: *F*_(1,__27)_ = 0.967, *p* = 0.334]. There was a significant difference observed between the two treatments [*F*_(1,__27)_ = 21.077, *p* < 0.001], indicating that female MIA-offspring buried fewer marbles than offspring from saline-injected mothers (Figure 5B). Means ± SD can be found in Supplementary Table 1.

### Three-Chamber Social Test

Social behavior was measured by determining the time offspring spent in the chambers containing the mesh cages. The social index was calculated using the time spent exploring mesh cages: $\frac{n\,o\,v\,e\,l\,c\,o\,n\,s\,p\,e\,c\,i\,f\,i\,c}{n\,o\,v\,e\,l\,c\,o\,n\,s\,p\,e\,c\,i\,f\,i\,c\,n\,o\,v\,e\,l\,o\,b\,j\,e\,c\,t}$. Means ± SD can be found in Supplementary Table 1.

**FIGURE 6:**
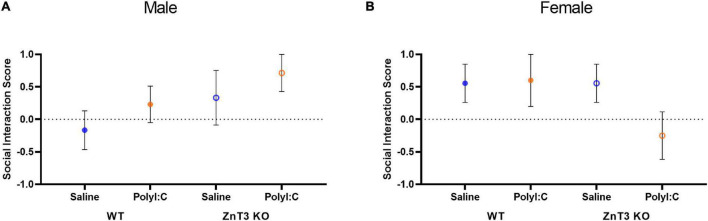
Social interaction assessed in the three-chamber social test. **(A)** In males, no significant difference was observed between treatment or genotype. **(B)** In females, no significant difference was observed between treatment or genotype. Error bars depict SEM.

In males, there was no significant difference between genotypes or treatments on the social index [main effect of genotype: *F*_(1,__34)_ = 0.140, *p* = 0.710; main effect of treatment: *F*_(1,__34)_ = 0.482, *p* = 0.492; genotype × treatment interaction: *F*_(1,__34)_ = 0.537, *p* = 0.468] (Figure 6A).

Likewise, in females, there was no significant difference between genotypes or treatments on the social index [main effect of genotype: *F*_(1,__27)_ = 0.055, *p* = 0.817; main effect of treatment: *F*_(1,__27)_ = 0.007, *p* = 0.936; genotype × treatment interaction: *F*_(1,__27)_ = 0.307, *p* = 0.584] (Figure 6B).

### Pre-pulse Inhibition

Means ± SD can be found in Supplementary Table 1.

**FIGURE 7:**
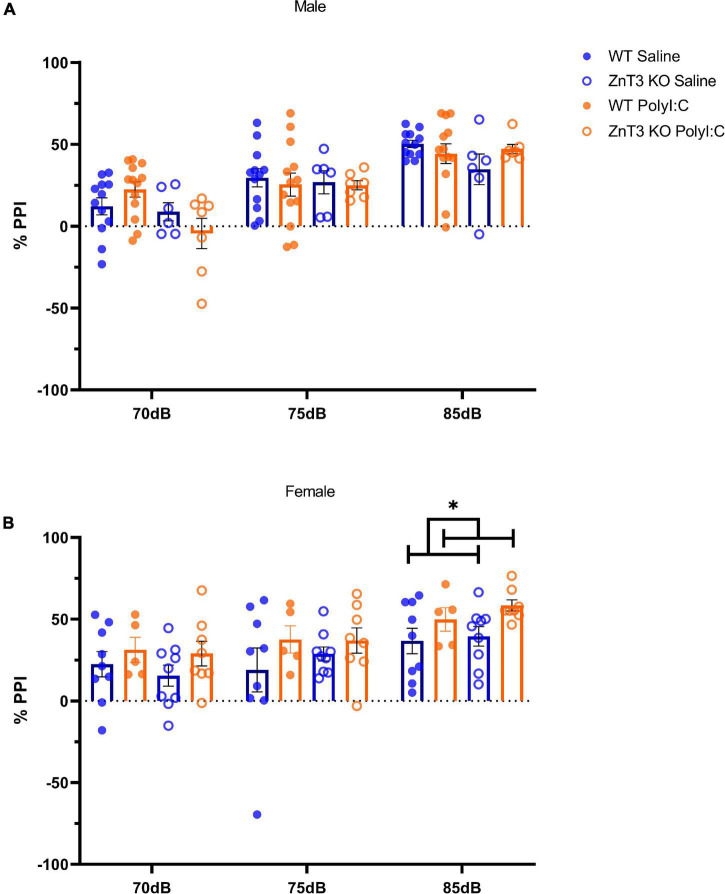
Sensory-motor gating assessment in the pre-pulse inhibition (PPI) test. **(A)** In males, no significant difference was observed between treatment or genotype. **(B)** Female wild-type (WT) maternal immune activation (MIA) offsprinxg showed greater inhibition and zinc transporter 3 knockout (ZnT3 KO) MIA offspring showed impaired inhibition compared to both WT and ZnT3 KO offspring of saline-injected mothers at 85 dB (PPI 20). Error bars depict SEM, *p < 0.05.

In males, the interaction between genotype and treatment at PPI 5 was close to significance, but this interaction was not significant at PPI 10 or 20 [PPI 5: *F*_(1,__34)_ = 3.487, *p* = 0.070; PPI 10: *F*_(1,__34)_ = 0.027, *p* = 0.871; PPI 20: *F*_(1,__34)_ = 2.655, *p* = 0.112]. A follow-up independent *t*-test did not identify a statistically significant difference between WT and ZnT3 KO offspring of saline-injected mothers at PPI 5 [*t*(16) = 0.398, *p* = 0.696] or ZnT3 KO MIA-offspring were less inhibited by the pre-pulse at 70 dB (PPI 5) compared to the WT MIA-offspring [*t*(18) = 2.904, *p* = 0.009, Bonferroni-corrected α = 0.008]. There was no significant main effect of treatment [PPI 5: *F*_(1,__34)_ = 0.058, *p* = 0.811; PPI 10: *F*_(1,__34)_ = 0.192, *p* = 0.664; PPI 20: *F*_(1,__34)_ = 0.339, *p* = 0.564] or genotype, with the exception of PPI 5, in which case ZnT3 KO male offspring were less inhibited by the pre-pulse at 70 dB (PPI 5) compared to WT offspring, [PPI 5: *F*_(1,__34)_ = 5.741, *p* = 0.022; PPI 10: *F*_(1,__34)_ = 0.054, *p* = 0.817; PPI 20: *F*_(1,__34)_ = 1.227, *p* = 0.276] (Figure 7A).

In females, there was no significant interaction between treatments and genotype on PPI 5, 10, or 20 [PPI 5: *F*_(1,__27)_ = 0.097, *p* = 0.758; PPI 10: *F*_(1,__27)_ = 0.286, *p* = 0.597; PPI 20: *F*_(1,__27)_ = 0.190, *p* = 0.666]. There was no significant main effect of treatment, except at PPI 20 where MIA-offspring had greater inhibition than offspring of saline-injected mothers [PPI 5: *F*_(1,__27)_ = 2.089, *p* = 0.160; PPI 10: *F*_(1,__27)_ = 1.867, *p* = 0.183; PPI 20: *F*_(1,__27)_ = 5.891, *p* = 0.022]. There was no genotype effect at any PPI intensity [PPI 5: *F*_(1,__27)_ = 0.360, *p* = 0.554; PPI 10: *F*_(1,__27)_ = 0.215, *p* = 0.646; PPI 20: *F*_(1,__27)_ = 0.750, *p* = 0.394] (Figure 7B).

## Discussion

The aim of this study was to determine the effects of prenatal immune challenge, MIA, in mice lacking vesicular zinc. We hypothesized that offspring of polyI:C-exposed dams would demonstrate an ASD- and SZ-like phenotype. Furthermore, we hypothesized that ZnT3 KO mice would show an enhanced ASD- and SZ-like phenotype compared to WT mice. Finally, we hypothesized that this phenotype would be more pronounced in male ZnT3 KO offspring compared to female ZnT3 KO offspring.

The results, summarized in Table 1, show that polyI:C-induced MIA at E12.5 leads to alterations in behavior of males that fall within one key diagnostic criterion for ASDs and SZ: communication. Anxiety-like, social, and repetitive-like behaviors remained unchanged. In females, MIA led to behavior alterations opposite to that of what was expected, changing one key diagnostic criteria for ASDs and SZ: repetitive-like behavior. Other measures of behavior such as communication, anxiety-like behavior, and social behavior, remained unchanged. As for sensorimotor gating measured in the PPI test, a deficit was observed in female (at 85 dB) ZnT3 KO offspring of polyI:C-injected mothers and greater inhibition was observed in female offspring of polyI:C-injected mothers (at 85 dB).

**TABLE 1:** Summary of behavioral statistically significant (*p* < 0.05) and marginally significant [*^#^*] (0.05 < *p* < 0.100) test results.

	Male	Female
**USVs**		
Number of calls (n)	Increased calls in MIA-offspring Increased calls in ZnT3 KO offspring	No difference
Length of calls (s)	No difference	No difference
Latency of call (s)	ZnT3 KO offspring were faster to emit their first call	No difference
USV min 1	Increased calls in ZnT3 KO offspring	No difference
USV min 2	Increased calls in MIA-offspring	No difference
USV min 3	Increased calls in MIA-offspring*^#^*	No difference
USV min 4	Increased calls in MIA-offspring*^#^*	No difference
**Open field**		
Distance traveled(m)	Decreased distance traveled in MIA- offspring*^#^*	Increased distance traveled in MIA- offspring
Total time spent in center (s)	No difference	No difference
Marble burying	Decreased marble burying in MIA- offspring*^#^*	Decreased marble burying in MIA- offspring
		Decreased marble burying in ZnT3 KO offspring
3-Chamber social test	No difference	No difference
**PPI**		
PPI5	Deficit in ZnT3 KO MIA-offspring	No difference
PPI10	No difference	No difference
PPI20	No difference	Greater inhibition in WT MIA- offspring
		Deficit in ZnT3 KO MIA-offspring

### Offspring of a Polyinosinic:Polycytidylic Acid Exposed Dams Demonstrated an Autism Spectrum Disorder- and Schizophrenia-Like Phenotype Only in Certain Behavior Tests

Maternal immune activation induction by polyI:C has been shown to produce an ASD- and SZ-like phenotype in offspring (Giovanoli et al., 2014; Grabrucker, 2014; Spann et al., 2018). Previous studies reported changes in USVs (increased or decreased calls), decreased distance traveled and decreased time spent in the center of the open field, increased marble burying, decreased social interaction, and deficits in PPI (Malkova et al., 2012; Schwartzer et al., 2013; Careaga et al., 2017; Kim et al., 2017). It is interesting that we observed different results in some of the tests. These difference could be due to the timing, dosage, and number of polyI:C injections (Kentner et al., 2018). Timing and dosage of polyI:C injection have been studied before, and the most common gestational points are E9, E12.5, and E17.

Changes in offspring behavior tests are observed when polyI:C is administered at any of the three gestational points; however, results vary depending on the tests performed. A study compared polyI:C administration at E9 and E17, and found opposite results, meaning that a deficit in one test at E9, was not significant at E17, and vice versa (Meyer et al., 2006b). This suggests that there may be different critical periods for the development of different brain systems/structures that underlie the different behaviors. Another study showed that polyI:C injection resulted in deficits in PPI and increased anxiety-like behavior at E12.5, but at E17, it resulted in decreased social interaction and time spent in the center of the open field (Meyer et al., 2006a,b; Ozawa et al., 2006; Hsiao et al., 2012; Reisinger et al., 2015).

Dosages of polyI:C used in previous studies vary from 2.5 to 20 mg/kg, and some studies have used multiple administrations rather than a single shot. The most commonly used dosage has been 20 mg/kg, and so far, it is the dose at which more differences have been observed in offspring. However, some studies have shown that lower doses produce more severe ASD- and SZ-like features in some, but not all, key symptoms. For example, 5 mg/kg of polyI:C administered three times during pregnancy produced extreme repetitive-like behavior and significantly lower sociability (Malkova et al., 2012). Aside from differences observed in behavioral outcomes in offspring, a dose-dependency has been observed in the maternal immune response, in which different doses activated different levels of pro-inflammatory cytokines (Meyer et al., 2005). This could influence the impact MIA has on the fetal brain and, consequently, the different behavioral outcomes that develop in adult offspring.

Although many studies have looked at MIA using different times, dosages, and number of polyI:C administrations, it is unclear how those differences truly impact the behavioral outcome of the offspring. This is due to a lack of replication in studies of MIA models (Kentner et al., 2018). Most studies use different assays, different timelines, different mouse models, and different purposes. One way that we could improve this is by increasing the transparency of the experimental design. For instance, in our experiment, many of the female breeders that did not deliver live pups were from the polyI:C-injected group. Furthermore, 72% of the dams from the saline group gave birth to viable litters, but only 49% of polyI:C-injected dams gave birth to viable litters. This could mean that the dosage of polyI:C we administered could have been too high for our mice. However, most studies of MIA models do not report losses. Many studies do not explain the reasoning for choosing a specific timeline. Doing so would allow others to better understand the approach and be better able to replicate the study. Providing exact measurements of the equipment or techniques used would also improve behavior testing in general.

Another important variable to consider is that many studies have tested offspring at different ages, which could explain the contrast between our results and what has been reported previously. Many of the published studies have used adolescent mice instead of adult mice. It is important to consider which symptoms we are evaluating to determine at what age we should test mice. For instance, if we are assessing ASD symptoms, we should test in early life stage and in adulthood. However, if we are testing SZ symptoms, which is diagnosed in adolescence and early adulthood in humans, we should test in adolescence and early adulthood to align with the clinical diagnosis we are trying to model (Kentner et al., 2018). The behavior tests should also align with the main purpose being studied. There is evidence that MIA models induce behavioral outcomes that are relevant across different neurological disorders, such as ASDs, SZ, depression, ADHD, bipolar disorder, and cerebral palsy (Canetta and Brown, 2012; Reisinger et al., 2015; Scola and Duong, 2017; Spann et al., 2018).

### Zinc Transporter 3 Knockout Mice Do Not Show Enhanced Autism Spectrum Disorder- and Schizophrenia-Like Phenotype

Contrary to what we had hypothesized, ZnT3 KO mice did not show a more severe ASD- and SZ-like phenotype than WT mice. A lack of differences between genotype could, perhaps, indicate that the ZnT3 KO mice compensated for the lack of zinc during development. It is also possible that the behavior tests used in this experiment did not require vesicular zinc signaling. Besides the study by Yoo et al. (2016), most studies that have looked at ZnT3 KO mice have found no difference or only mild differences in behavior tests between KO and WT genotypes (Cole et al., 2001; Martel et al., 2011; Thackray et al., 2017).

In the introduction, we mentioned a study that found an ASD-like phenotype in male ZnT3 KO mice (Yoo et al., 2016). Since we did not see the same results they did, it is worth exploring what was different in our experimental approaches. The first possibility that could explain the differing results, is that Yoo et al. (2016) ran behavior tests in mice that were 4–5 weeks of age. However, we ran our behavior tests between 60 and 75 days of age (8–9 weeks of age), except for USVs, which we measured at P9. This would suggest that ZnT3 KO offspring show big differences at a younger age, but these effects are short-lasting and go away a few weeks later. Another possible explanation would be that we tested behaviors in a different order than they did and used different tasks; they started with the three-chamber social test, then marble burying, with the open field or reciprocal social interaction tests last. It also appears that they conducted the three-chamber social test slightly differently: one side chamber had a conspecific and the other side (and middle chamber) were empty, then they placed the same conspecific in one side and a new conspecific on the other side, leaving the middle chamber empty (Yoo et al., 2016). Having two conspecifics, rather than one conspecific and a novel inanimate object, such as the one presented here, could possibly be evaluating different features. The approach used by Yoo et al. (2016) is testing the curiosity-like behavior of a mouse to a novel conspecific compared to how much time it spends exploring the other conspecific that is no longer new. In our approach, we were looking at the preference between socializing with a novel conspecific or exploring a novel inanimate object.

Previous studies that used the ZnT3 mouse model to study behavioral outcomes, observed no difference between WT and ZnT3 KO mice for the time spent in the center or the distance traveled in the open field test (Cole et al., 2001; Martel et al., 2010; Thackray et al., 2017). ZnT3 KO mice also did not show social or PPI deficits compared to WT mice (Cole et al., 2001; Martel et al., 2011; Thackray et al., 2017). Based on these observations, our results are similar in that no differences were observed between ZnT3 KO mice and WT mice.

### Autism Spectrum Disorder- and Schizophrenia-Like Phenotype Is Not More Pronounced in Male Zinc Transporter 3 Knockout Compared to Female Zinc Transporter 3 Knockout

We observed no significant difference between male and female ZnT3 KO mice. However, male offspring had more statistically significant differences than females did in the behavior assays.

It is possible that MIA, in general, affects females differently than males. Most studies have investigated male offspring and the studies that looked at both sexes either found no sex differences or only males showed significant ASD- and SZ-like phenotype (Grabrucker et al., 2016; Hui et al., 2018; Coiro and Pollak, 2019; Lins et al., 2019; Gogos et al., 2020). It is well known that clinical diagnosis of ASD and SZ is more common in males than females (Public Health Agency of Canada, 2018, 2020). This could potentially be due to differences in the severity of symptoms related to ASD and SZ, where female symptoms are more subtle than in males. Therefore, it is not surprising that our results show differences in key features of ASD and SZ in males, but not females. A reason we may be seeing sex differences in our results, namely, how males show more ASD- and SZ-like features than females, could be due to estrogen. A relationship between estrogen and ZnT3 has been shown, in which case, higher levels of estrogen reduce ZnT3 levels (Lee et al., 2004). Estrogen has also been shown to influence inflammatory response; that is, lower levels of estrogen increase inflammation (Monteiro et al., 2014).

### Autism Spectrum Disorder- and Schizophrenia-Like Phenotype Is Not More Pronounced in Zinc Transporter 3 Knockout Offspring of Polyinosinic:Polycytidylic Acid Exposed Dams

We hypothesized that MIA prenatal exposure would affect the brain in the fetus causing important alterations that could interact with loss of ZnT3 later in life. Our results suggest that there is no interaction between MIA exposure and loss of vesicular zinc. Since we observe deficits in some PPI results, it is possible that the interaction between MIA and ZnT3 KO is more relevant to a schizophrenic model. This could be further explored by testing other symptoms of SZ-like behavior such as locomotor activity in response to psychotomimetic drugs (e.g., ketamine), working memory (e.g., T-maze working memory task), and spatial navigation (e.g., Morris water task) (Powell and Miyakawa, 2006).

Previous studies have shown that prenatal injection of polyI:C affect maternal care behavior, and an aspect we did not measure in this study (Ronovsky et al., 2017; Berger et al., 2018). Consequently, changes in maternal care have been shown to greatly impact offspring behavior (Champagne et al., 2008; Ronovsky et al., 2017; Berger et al., 2018). A possible way to control for maternal care effect would be to use cross-fostering design and to include maternal care measures in the experimental design, such as nest building, licking of pups, and pup retrieval (Richetto et al., 2013; Ronovsky et al., 2017; Berger et al., 2018).

## Conclusion

For this study, a lack of vesicular zinc did not produce offspring that were more susceptible to developing ASD- and SZ-like features in all the behavior assays performed in this experiment. We observed an ASD- and SZ-like phenotype in male offspring of polyI:C-injected dams, but not in female offspring.

It is important to keep in mind that environmental stressors and genetic mutations do not lead to NDDs. Rather, these events increase the risk of changes in brain morphology and behavior. Not all offspring exposed to MIA will develop an NDD later in life.

## Data Availability Statement

The raw data supporting the conclusions of this article will be made available by the authors, without undue reservation.

## Ethics Statement

The animal study was reviewed and approved by the Life and Environmental Sciences Animal Research Committee, University of Calgary.

## Author Contributions

KS collected and analyzed the data and wrote the original draft of the manuscript. ST, AW, and NN helped to carry out the experiment. CC and SR completed the initial and pilot experiments. KS, ST, and RD helped to supervise the project and design the experiment. RD reviewed and edited the manuscript. All authors contributed to the article and approved the submitted version.

## Conflict of Interest

The authors declare that the research was conducted in the absence of any commercial or financial relationships that could be construed as a potential conflict of interest.

## Publisher’s Note

All claims expressed in this article are solely those of the authors and do not necessarily represent those of their affiliated organizations, or those of the publisher, the editors and the reviewers. Any product that may be evaluated in this article, or claim that may be made by its manufacturer, is not guaranteed or endorsed by the publisher.
