# Supplementary material for: Lack of Vesicular Zinc Does Not Affect the Behavioral Phenotype of Polyinosinic:Polycytidylic Acid-Induced Maternal Immune Activation Mice
Source: Front Behav Neurosci. 2022 Feb 22;16:769322. doi: 10.3389/fnbeh.2022.769322 (PMC8902171; doi:10.3389/fnbeh.2022.769322)
Supplement: Supplementary file 1 [file Table_1.pdf]

Lack of vesicular zinc does not affect the behavioral phenotype of poly (I:C)-induced MIA mice

Supplemental Table 1 Additional statistical measures of behavioural assessments. Statistics are reported as mean ± standard deviation

	WT Saline		WT PolyI:C		ZnT3 KO Saline		ZnT3 KO PolyI:C	
**USVs**	Female (*n* = 9)	Male (*n* = 12)	Female (*n* = 5)	Male (*n* = 13)	Female (*n* = 9)	Male (*n* = 7)	Female (*n* = 8)	Male (*n* = 7)
Number of calls (n)	282.00 ± 169.881	297.08 ± 137.200	264.80 ± 99.535	358.77 ± 94.407	295.78 ± 161.794	362.83 ± 160.296	333.00 ± 167.244	483.71 ± 105.696
Length of calls (s)	0.069 ± 0.008	0.075 ± 0.011	0.069 ± 0.010	0.075 ± 0.009	0.071 ± 0.010	0.074 ± 0.011	0.075 ± 0.007	0.071 ± 0.005
Latency of call (s)	10.600 ± 14.762	11.846 ± 16.033	1.415 ± 1.570	10.627 ± 8.549	5.228 ± 10.029	3.613 ± 3.623	7.787 ± 8.130	3.031 ± 3.915
USV min 1	65.11 ± 46.482	68.08 ± 40.469	81.60 ± 58.943	69.23 ± 46.004	82.22 ± 44.432	110.33 ± 49.127	72.13 ± 59.237	141.43 ± 20.743
USV min 2	70.78 ± 50.719	82.83 ± 37.472	56.80 ± 40.295	97.08 ± 25.115	71.33 ± 49.115	92.83 ± 40.578	87.13 ± 51.546	133.29 ± 33.758
USV min 3	73.78 ± 44.614	75.67 ± 34.036	71.20 ± 37.851	98.69 ± 33.182	76.22 ± 44.280	86.50 ± 46.976	86.88 ± 29.821	115.14 ± 41.607
USV min 4	72.33 ± 48.234	62.17 ± 38.560	55.20 ± 30.614	93.77 ± 29.204	66.00 ± 55.064	73.17 ± 51.098	86.75 ± 46.665	93.86 ± 46.395
**Open field**								
Distance travelled (m)	39.986 ± 8.130	48.351 ± 15.548	58.137 ± 19.793	41.720 ± 16.307	43.818 ± 9.095	47.829 ± 13.893	43.782 ± 10.781	35.761 ± 5.504
Total time spent in centre (s)	62.378 ± 26.058	78.396 ± 26.016	63.576 ± 21.241	70.747 ± 35.592	65.089 ± 27.204	77.509 ± 28.596	77.989 ± 30.843	97.480 ± 62.557
**Marble burying**	0.842 ± 0.084	0.900 ± 0.100	0.685 ± 0.084	0.873 ± 0.056	0.864 ± 0.064	0.888 ± 0.086	0.763 ± 0.074	0.814 ± 0.097
**3-Chamber social test**	-0.33 ± 1.000	-0.17 ± 1.030	0.20 ± 1.095	0.23 ± 1.013	-0.11 ± 1.054	0.33 ± 1.033	0.00 ± 1.069	0.71 ± 0.756
**PPI**								
PPI5	22.387 ± 23.204	12.143 ± 17.919	31.114 ± 17.273	22.369 ± 16.761	15.385 ± 19.356	8.820 ± 13.636	28.891 ± 21.298	-4.431 ± 24.514
PPI10	18.890 ± 40.303	29.535 ± 19.331	37.552 ± 18.591	25.398 ± 25.390	28.692 ± 12.783	26.808 ± 17.239	36.861 ± 21.834	24.928 ± 7.410
PPI20	36.632 ± 23.325	50.179 ± 7.728	49.769 ± 16.067	44.268 ± 21.660	39.466 ± 17.975	34.734 ± 22.925	58.360 ± 9.434	47.213 ± 7.123
